# “Girls Questionnaire for Autism Spectrum Condition” as a screening protocol - pilot study

**DOI:** 10.1192/j.eurpsy.2025.2421

**Published:** 2025-08-26

**Authors:** M. M. N. Matsumoto, A. L. G. D. O. Araujo, D. R. Molini-Avejonas

**Affiliations:** 1 University of Sao Paulo, Sao Paulo, Brazil

## Abstract

**Introduction:**

Currently, we see an increase in the identification and diagnosis of girls with Autism Spectrum Disorder (ASD) over the years, but where are these girls, now adult women, in the Brazilian population? There are barriers that delay and prevent early diagnosis and intervention in women with ASD, such as active attempts to camouflage or mask the signs of ASD in challenges related to social situations. It is known that people with ASD have high rates of depression, self-harm, and suicidal thoughts, which are exacerbated by difficulties in accessing treatment, professional, and family support. In this sense, obtaining the correct diagnosis and treatment for women with ASD is a challenge, as the measures developed and validated are primarily based on male samples and may not be sensitive to the female autism phenotype. With this in mind, the use of screening protocols is a tool that could minimize these impacts, improve quality of life, and drastically change the referral pathways for adult women with ASD.

**Objectives:**

To present the results of the use of the Girls Questionnaire for Autism Spectrum Condition (GQ-ASC) protocol.

**Methods:**

The screening questionnaire, Girls Questionnaire for Autism Spectrum Condition (GQ-ASC), developed by Brown et al., 2020, was used. In this study, a freely translated version was used, as there is no cross-cultural adaptation to Brazilian Portuguese. The questionnaire was sent via an online form, where participants who accepted the Informed Consent Form had access to the GQ-ASC. The study was approved by the Research Ethics Committee under protocol 65890317.9.0000.0065. The questionnaire consists of 21 items, assessed on a 4-point Likert scale (1=Strongly Disagree, 2=Disagree, 3=Agree, 4=Strongly Agree) that evaluate specific clinical characteristics of ASD presentation in adult females across five dimensions: imagination and play, camouflage, sensory sensitivity, socialization, and interests. At the end, the scores are summed, with a total score above 56 indicating a high level of autistic traits, with 80% sensitivity in the author’s studies.

**Results:**

31 women with self-reported ASD diagnoses responded to the questionnaire. The average age was 24.09 years, 80.64% identified as white, 61.29% reported incomplete higher education, and 22.58% postgraduate education, 25.80% identified as bisexual, and 25.80% as heterosexual. For the GQ-ASC questionnaire, 83.87% of the participants scored above 56, with an average final score of 64.12 and a standard deviation of 7.95.

**Conclusions:**

The results of the pilot study demonstrated that the final score of the GQ-ASC corroborates the self-reported diagnosis of ASD in adult women. The GQ-ASC can be considered a screening tool for this population, requiring cross-cultural adaptation to Brazilian Portuguese and validation so that more research and refinement of this tool can improve the quality of life for these women.

**Disclosure of Interest:**

None Declared

